# Factors Influencing Microbial Contamination of Groundwater: A Systematic Review of Field-Scale Studies

**DOI:** 10.3390/microorganisms12050913

**Published:** 2024-04-30

**Authors:** Francesco Bagordo, Silvia Brigida, Tiziana Grassi, Maria Clementina Caputo, Francesca Apollonio, Lorenzo De Carlo, Antonella Francesca Savino, Francesco Triggiano, Antonietta Celeste Turturro, Antonella De Donno, Maria Teresa Montagna, Osvalda De Giglio

**Affiliations:** 1 Department of Pharmacy-Pharmaceutical Sciences, University of Bari Aldo Moro, Via Orabona 4, 70125 Bari, Italy; francesco.bagordo@uniba.it; 2 Department of Experimental Medicine, University of Salento, Via Monteroni 165, 73100 Lecce, Italy; silvia.brigida@unisalento.it (S.B.); antonella.dedonno@unisalento.it (A.D.D.); 3 National Research Council of Italy (CNR), Water Research Institute (IRSA), Via F. De Blasio, 5, 70132 Bari, Italy; mariaclementina.caputo@cnr.it (M.C.C.); lorenzo.decarlo@cnr.it (L.D.C.); celeste.turturro@irsa.cnr.it (A.C.T.); 4 Interdisciplinary Department of Medicine, University of Bari Aldo Moro, Piazza G. Cesare 11, 70124 Bari, Italy; francesca.apollonio@uniba.it (F.A.); francesco.triggiano@uniba.it (F.T.); mariateresa.montagna@uniba.it (M.T.M.); osvalda.degiglio@uniba.it (O.D.G.); 5 Hygiene Section, Azienda Ospedaliero Universitaria Policlinico di Bari, Piazza Giulio Cesare 11, 70124 Bari, Italy; antonellasavino8@yahoo.it

**Keywords:** microbial contamination, groundwater, field studies, public health, geological settings, environmental factors

## Abstract

Pathogenic microorganisms released onto the soil from point or diffuse sources represent a public health concern. They can be transported by rainwater that infiltrates into subsoil and reach the groundwater where they can survive for a long time and contaminate drinking water sources. As part of the SCA.Re.S. (Evaluation of Health Risk Related to the Discharge of Wastewater on the Soil) project, we reviewed a selection of field-scale studies that investigated the factors that influenced the fate of microorganisms that were transported from the ground surface to the groundwater. A total of 24 studies published between 2003 and 2022 were included in the review. These studies were selected from the PubMed and Web of Science databases. Microbial contamination of groundwater depends on complex interactions between human activities responsible for the release of contaminants onto the soil, and a range of environmental and biological factors, including the geological, hydraulic, and moisture characteristics of the media traversed by the water, and the characteristics and the viability of the microorganisms, which in turn depend on the environmental conditions and presence of predatory species. Enterococci appeared to be more resistant in the underground environment than thermotolerant coliforms and were suggested as a better indicator for detecting microbial contamination of groundwater.

## 1. Introduction

The occurrence of microbiological contamination in freshwater resources used for drinking or irrigation purposes represents both environmental and public health issues that give rise to great concern. At present, the entire global population is still not guaranteed access to a clean water supply and adequate water facilities [1]. Moreover, in recent decades, the global demand for water for human needs has increased because of demographic growth [2]. At the same time, the availability of high-quality water resources has decreased because of the increase in anthropic pressures, mainly related to urbanization, industrialization, agricultural and livestock practices, and the effects of climate change. The interactions of these factors have increased the risks of contamination from human pathogens and the diffusion of waterborne diseases.

People who are exposed to contaminated water can experience adverse health effects, such as *E. coli* infections, diarrhoea, giardiasis, cholera, typhoid fever, salmonellosis, hepatitis A, and polio. It has been estimated that about one million people, of whom 395,000 are children under 5 years old, die every year because of diarrhoea epidemics that result from poor sanitation and the use of unsafe water in domestic settings [3]. 

Of the water resources that can be used for human purposes, including irrigation and drinking water, groundwater is considered to be valuable since it is protected from pollutants released on the surface by the layers of soil above it [4]. However, if anthropogenic pressures are particularly heavy and/or groundwater is highly vulnerable, these resources may be affected by microbiological pollution [5]. The microbiological contamination of groundwater can be traced back to the presence of point or diffuse contamination sources, which are mainly represented by wastewater, livestock manure applications on soils, and animal excreta. 

Wastewater represents the final stage of the water use cycle. Before being returned to the environment, wastewater must be treated in wastewater treatment plants (WWTPs) in order to reduce the organic load and the infectious hazards. The effluents from these plants are discharged onto the ground or directly into surface water bodies. Because many urban and arid environments experience water scarcity, local populations have to rely on alternative water resources that are often represented by treated wastewater which may be discharged into the hydrographic network to restore the environmental baseflow of ephemeral rivers [6,7,8,9], enhance groundwater recharge processes [10], or used as irrigation or drinking water sources [11]. Therefore, the release of raw or improperly treated wastewater into the environment could spread pathogens into water supply sources and have effects on human health [12]. Additionally, as the climate changes, we expect that extreme weather events will occur more frequently worldwide, causing accidental wastewater overflows [13,14] and the consequent spread of infectious hazards [15,16,17,18]. Because of its nutrient content, livestock manure is widely used to improve soil and to make it more productive for growing crops. However, the presence of different types of pathogens in manure represents a threat to public health, as they may be transported by rainfall or irrigation water from the soil through the vadose zone to the groundwater [19,20]. 

Thanks to hydrological drivers such as rainfall or irrigation events, microorganisms contained in animal dejections may be mobilized in runoff that infiltrates through the superficial soil and the unsaturated zone into the groundwater [19].

The fate of pathogenic microorganisms that are released from different polluting sources in soils and aquifers is mainly contingent on the conditions that influence their survival in these environments. Additionally, the geological and hydrogeological settings can affect the mobilization of microorganisms, depending on the physical properties and geological features of the media that the water has passed through and the type of aquifer [21,22,23,24]. Since the 1990s, a range of factors that participate in the contamination of environmental matrices have been investigated in both field-scale and laboratory-scale experiments by several authors.

Specifically, in this paper, we present a systematic review of published field-scale studies that have investigated the factors that can influence the transport and fate of microorganisms from the ground surface to groundwater. This study was part of the SCA.Re.S. (Evaluation of Health Risk Related to the Discharge of Wastewater on the Soil) project, which was funded by the Apulia Regional Government to evaluate the risk of contaminating groundwater by discharging effluents from wastewater treatment plants in the draining trench.

## 2. Materials and Methods

In this study, we followed the guidelines in the Preferred Reporting Items for Systematic Reviews and Meta-Analyses (PRISMA) statement [25]. Literature published from 2003 to 2022, identified using the PubMed/Medline and Web of Science (WOS) databases, was reviewed to examine the extent, range, and nature of recent field-scale investigations into the fate of microorganisms of human concern and their transport to groundwater through the unsaturated zone.

The search was carried out on 31 March 2023. We entered “water”, “groundwater”, “wastewater”, “infiltration”, “leaching”, “transport”, “microorganisms”, “bacteria”, “virus”, “pathogens”, “critical zone”, “vadose zone”, “saturated”, and “unsaturated” as keywords in different combinations in the “title” and “abstract” search options. The results of the individual searches were imported into the Rayyan (http://www.rayyan.ai, accessed on 31 March 2023) screening tool for subsequent analysis.

First, any duplicate records were removed from the search. Then, the records were independently screened by four authors (F.B., S.B., T.G. and M.C.C.) through the revision of the information contained in the title and abstract, according to the following inclusion criteria: (1) the study was published between 2003 and 2022; (2) the research was reported in a full-text article and published in English in a peer-reviewed journal; (3) the study was carried out at the field scale; and (4) the target microorganisms were allochthonous bacteria and/or viruses. 

Records were excluded if they were (1) conference contributions or abstracts; (2) reviews, meta-analyses, letters, notes, reports, commentaries, or editorials; (3) studies of subsoil microbial communities; (4) studies that used microorganism surrogates (colloids, microspheres, chemical tracers, etc.); (5) based on artificial substrates; (6) lacking (either completely or partially) in microbiological data; or (7) were descriptions of study protocol, study design, methodology, or modelling based on previous studies, with the exception of case studies.

The four authors then read the full-text versions of the eligible articles to identify the relevant studies to include in the review. Any disagreements about eligibility between the reviewers were resolved by discussion.

The relevant information (country in which the research was performed, study design, type of media investigated, and target microorganism) as well as the main findings of each study were reported in tables.

Finally, factors associated with the survival, growth, or transport of microorganisms in the subsurface were grouped into five categories (geological and hydrogeological setting, hydraulic properties, pollution load, environmental factors, and microbial characteristics) and discussed. The strengths and limitations of the analysed literature were also discussed.

## 3. Results

### 3.1. Study Selection

**Figure 1:**
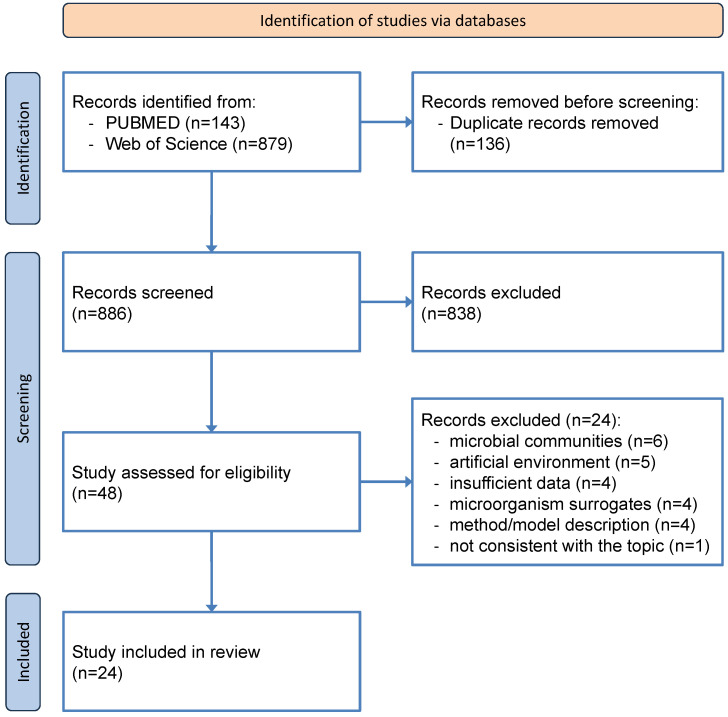
PRISMA flow diagram describing the study identification and selection process.

The process used to select the studies for the review is reported in a flow chart (Figure 1). After searching the Pubmed and WOS databases, removing duplicates, and selecting records, 48 studies met the inclusion criteria and were assessed for eligibility.

Twenty-four studies were excluded after the full-text paper had been read because six had subsoil microbial communities as their subject [26,27,28,29,30,31], five were conducted in artificial environments [32,33,34,35,36], four had insufficient microbiological data [37,38,39,40], four used microorganism surrogates [41,42,43,44], four were descriptions of methodologies or models [45,46,47,48], and one was not consistent with the topic of the review [49].

The information in the twenty-four studies that were selected for the review [50,51,52,53,54,55,56,57,58,59,60,61,62,63,64,65,66,67,68,69,70,71,72,73] was then grouped into six main categories: Study, Location, Contamination source, Environmental domain, Target microorganisms, and Study design, as shown in Table 1. 

### 3.2. Characteristics of the Studies

#### 3.2.1. Geographical Areas

**Figure 2:**
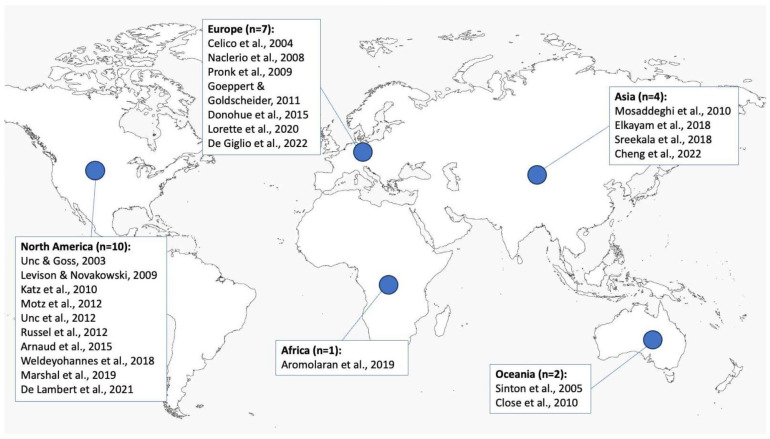
Distribution of the studies selected for the review by geographical area North America: [50,54,58,60,61,62,64,67,69,71]; Europe: [51,53,55,59,63,70,72]; Africa: [68]; Oceania: [52,57]; Asia: [56,65,66,73].

Figure 2 shows the geographical distribution of the study sites of the selected studies. Most of these field-scale studies for the period of interest (from 2003 to 2022) were carried out in North America (*n* = 10) and Europe (*n* = 7), followed by Asia (*n* = 4), Oceania (*n* = 2), and Africa (*n* = 1).

#### 3.2.2. Contamination Sources

The study sites were affected by both diffuse and point contamination sources. With regard to diffuse sources, many authors investigated the pressures from agricultural and livestock practices. In particular, four studies [50,56,61,64] described the transport of microorganisms from manure applied to the soil, while five studies [51,53,54,57,59] considered cattle pastures. 

Some authors focused their research on microbial transport in subsoil caused by infiltration following rainfall [55,66], while others focused on the infiltration of surface runoff [70] or urban stormwater [71]. Finally, polluted sands [62] and polluted seawater [73] were considered as contamination sources for coastal aquifers.

Effluents from WWTPs, including on-site systems (OSWTS), as a potential point sources of groundwater contamination, were also discussed [52,60,63,65,72] as well as the impacts of untreated wastewater [67,69], septic tanks [58], and a municipal solid waste (MSW) dumpsite [68]. 

#### 3.2.3. Environmental Domain

The studies were carried out on different media. We distinguished between studies of soil and aquifers. The latter was further divided into “unsaturated” and “saturated” zones, depending on the condition in which the investigation was carried out. Furthermore, the aquifer was classified as “porous”, “fractured”, or “karst”, depending on the rock characteristics constituting the aquifer. 

Most of the studies focused on the saturated and unsaturated zones of aquifers but two focused on soils [50,56]. Of the studies that investigated porous aquifers, five examined the saturated portion [52,59,63,67,73]; two examined the unsaturated domain [57,60], and four examined both the saturated and unsaturated domain [61,62,65,71].

Five studies investigated the saturated portion of fractured aquifers [51,53,54,68,69]. Karst aquifers were investigated in three studies; of these, two focused on the saturated portion [58,70] and one focused on the unsaturated zone [55]. 

Some of the studies compared different situations. For example, one study compared the behaviour of microorganisms in porous and fractured aquifers [66]. Another one compared the fate of microorganisms in a porous aquifer and a karst aquifer [72]. Finally, one study involved an aquifer that comprised an unsaturated porous zone and a saturated fractured zone [64].

#### 3.2.4. Target Microorganisms

Bacterial species or groups were considered in nineteen studies [50,51,53,54,55,56,57,59,60,61,62,63,64,66,67,68,69,70,73] and five papers reported the behaviour of both bacteria and viruses [52,58,65,71,72].

With regard to bacterial species, the environmental fate of *E. coli* was widely studied [50,52,54,55,56,57,59,60,63,64,65,66,67,69,70,71,72,73]. *Bacillus subtilis* endospores [52,57], *Campylobacter jejuni* [57], and *Clostridium perfringens* [72] were also reported. Many studies focused on the observation of faecal indicator bacteria such as total coliforms [50,63,64,66,68,69,70,71], faecal coliforms (including thermotolerant coliforms) [51,52,53,57,58,65,66,68], and enterococci (including faecal streptococci and *Enterococcus* spp.) [51,53,55,59,61,62,65,70,72].

With regard to viruses, De Giglio et al. [72] focused their research on enteric viruses, including adenovirus, norovirus genogroup I and II, enterovirus, hepatitis A virus, hepatitis E virus, and rotavirus. Elkayam et al. [65], on the other hand, focused on adenovirus, enterovirus, norovirus, and parechovirus. Additionally, Eeteroviruses were investigated by Katz et al. [58] and group A rotaviruses were investigated by de Lambert et al. [71]. Finally, Sinton et al. [52] considered the behaviour of F-RNA phages and somatic coliphage ØESR1.

#### 3.2.5. Study Design

The fate of microbial contaminants released by human activities was assessed by monitoring their concentration after they had flowed through the subsoil layers. In most cases [52,54,57,58,61,62,63,64,65,66,67,68,69,71,72,73], the output was groundwater, sampled through monitoring wells. Additionally, spring water [51,53,70], water dripping from a fracture [55], and recovery water [65] were also sampled. 

Where feasible, the sources of contamination were monitored. This was mainly performed for point sources [58,60,65,67,68,72] while the concentration or presence of microorganisms in diffuse sources was evaluated in manure applied to soils [50,61,64], in seawater that infiltrated sand [62], and in a rainwater collection basin [71]. 

Sinton et al. [52] evaluated the horizontal attenuation of microbial contamination in a saturated porous aquifer by monitoring a well located beneath the pollution source and a well situated 132 m downgradient.

To allow the behaviour of microorganisms in the subsoil to be defined in detail, the study designs involved transport experiments performed by collecting the leachate using lysimeters [56,57,60,62,71] or collection tubes [68], or (3) collecting soil samples with a corer [50,56,58,61]. 

In five studies [51,53,57,61,62], the results of field tests were integrated with the results of laboratory tests.

### 3.3. Factors Influencing Microbial Growth, Survival, or Transport

Table 2 shows a comparison between the concentrations of microorganisms or their frequencies detected in the contamination sources and groundwater reported by the selected studies. In general, the presence of microbial contaminants was dramatically lower in the monitored outlets compared to the sources. Various factors could influence the survival or attenuation and transport of microorganisms in the subsoil. Many of them were considered in the literature included in this review and were grouped in the following categories: land-use activities, environmental factors, hydraulic properties of the media through which the water moves, geological and hydrogeological settings, and microbial characteristics. The main results from each study are discussed below and are summarized in Table 3.

#### 3.3.1. Land-Use Activities

Microbial contaminants may be released onto the soil through anthropic activities, and then percolate into the subsoil and contaminate groundwater. Marshall et al. [69] highlighted that wastewater derived from a sparse distribution of small, aging septic systems posed a serious threat to groundwater in a shallow fractured aquifer with a thin unsaturated zone, because the contaminants were rapidly transported through the fractures with little attenuation.

On examining the leachate from a MSW dumpsite, Aromolaran et al. [68] detected a variety of microorganisms that were responsible for the microbial contamination of the fractured aquifer. These microorganisms included bacteria from the enteric flora of humans and warm-blooded animals, presumably sourced from faecal materials in the waste.

Goeppert and Goldscheider [59] found that the temporal variability in faecal indicator bacteria in the springs from porous aquifers reflected the different intensities of cattle grazing in the aquifer recharge areas.

Close et al. [57] compared variations in microbe transport through the soil and vadose zone to groundwater from pasturing of dairy cows, and observed that spray irrigators resulted in less transport of microbes than other irrigation systems. 

Donohue et al. [63] demonstrated that the groundwater in a poorly productive aquifer sustained severe contamination close to an area where microbiologically contaminated effluent from an OSWTS had percolated through a thin layer of glacial till subsoil.

#### 3.3.2. Environmental Factors

The growth, survival, and transport of microorganisms in the subsoil were deemed to be influenced by many meteorological and environmental conditions. As rainwater is an important driver for runoff and the leaching of microorganisms, rainfall events were frequently associated with increased risks of microbial contamination of groundwater in porous aquifers [57,66,71], karst aquifers [55,70], and fractured aquifers [51,54,66].

Cheng et al. [73] highlighted that the environmental factors in a beach groundwater system influenced *E. coli* removal and attenuation. In particular, they observed that the removal of *E. coli* from a beach aquifer was strongly influenced by environmental factors such as the tidal level, water salinity and temperature, air temperature, wind speed, and rainfall. Specifically, the beach aquifer removed *E. coli* from the surf zones most of the time, except during periods of very high tidal levels and heavy rainfall, when the water salinity decreased and bacterial survival increased as a consequence.

Motz et al. [60] observed that the metabolic rates of both *E. coli* and their predators were influenced by seasonal variations in temperature. They found that the *E. coli* survival was high in the winter season but was lower in the warmer temperatures during the summer period, when the bacteria removal was promoted by an increase in predation by protozoa.

#### 3.3.3. Hydraulic Properties

Studies of aquifers affected by microbial pollution in manured areas demonstrated that the hydraulic properties of the media the water passed through, i.e., the soil and/or rock, influenced the mobilization of microorganisms through the unsaturated zone, by conditioning the groundwater contamination. For example, Unc and Goss [50] observed that high moisture levels facilitated microbial transport from surficial polluted soils towards the groundwater. Specifically, the bacterial transport rate was higher in saturated conditions than unsaturated conditions, and the rate decreased as the moisture level decreased [56]. Finally, Unc et al. [61] concluded that intensive irrigation and abundant rainfall promoted the rapid transport of bacteria in the surficial layers of soil rather than through the deep unsaturated zone.

#### 3.3.4. Geological and Hydrogeological Settings

Many studies considered that the fate of microbiological contaminants in the subsoil was influenced by the geological or hydrogeological setting. Here, most of the research focused on the role of the unsaturated zone.

In their studies, Elkayam et al. [65] and Weldeyohannes et al. [67] stressed that the thickness of the unsaturated zone influenced the removal of faecal bacteria and viruses from contaminated water that infiltrated the subsoil. Elkayam et al. [65] found that a 30–40 m thick vadose zone, with no preferential pathways, was an effective barrier between an infiltration basin that received secondary effluents from a WWTP and the underlying saturated zone, and that this vadose zone had a sufficient ability to disinfect the groundwater, so that it could be used for irrigating crops for unrestricted raw consumption. Weldeyohannes et al. [67] observed that the concentrations of *E. coli* that originated in effluent from an OSWTS decreased dramatically in shallow groundwater monitoring wells when the thickness of the unsaturated zone, comprising homogeneous glacial deposit with no macropores or fractures, exceeded 0.9 m.

Arnaud et al. [64] detected groundwater contamination by *E. coli* from livestock manure applications on fields despite a 12 m thick unsaturated zone comprising coarse and heterogeneous glacial sediments. They assumed that preferential pathways, resulting from (1) macropores, (2) sediment heterogeneity, (3) fractures, (4) local broad swales that generated depression-focused infiltration, and (5) fractures in the underlying bedrock, facilitated faster transport of contaminated water from the surface to the groundwater.

In their study, Sinton et al. [52] found that the transport velocities of WWTP effluents containing enteric microorganisms used to irrigate soil strips and subsequent groundwater contamination were mainly attributable to the presence of macropores within the unsaturated zone.

Unc and Goss [50] investigated bacterial transport in a silty loam soil and a sandy loam soil that were treated with liquid swine manure and solid cow manure. Although the total porosity was greater in the sandy loam soil, they found that the groundwater in the silty loam soil was more frequently contaminated than that in the sandy loam soil. They also concluded that the presence of macropores had more influence on the transport of bacterial suspensions than the moisture content of the soil.

Russel et al. [62] found that enterococci could be mobilized and microorganisms could be transported from naturally contaminated beach sands to the groundwater when seawater intermittently infiltrated through the sands and the unsaturated zone during high tides.

Katz et al. [58] studied how a karst aquifer was contaminated by septic tank effluent, and detected high levels of faecal indicators and enteric viruses in the shallowest portion of a limestone karst aquifer, overlayed by a thin layer of sands and clays.

Finally, De Giglio et al. [72] compared the fate of bacteria and viruses contained in the effluents of two WWTPs that discharged to dispersing trenches overlying a porous vadose zone and karst vadose zone, respectively. They observed that the microbial concentration decreased more in the porous vadose zone than in the karst one.

#### 3.3.5. Microbial Characteristics

As reported in Table 2, the occurrence of microorganisms in groundwater exhibits considerable variability among different microbial species or groups, even when searched simultaneously. This variability may be attributed to the varying concentrations they had in the contamination sources. In general, total coliforms, faecal coliforms, and, among bacterial species, *E. coli* were released in greater quantities than other bacterial indicators. However, in some cases, it was possible to detect a different rate of decrease among different microbial contaminants. The studies selected for this review showed that the nature of the microorganisms, including their morphological and biological characteristics, affected their survival in or transport through the subsoil and the different interactions they established with the biotic and abiotic components.

Close et al. [57] observed higher detection rates of coliform bacteria compared with *Campylobacter* in both transport experiments and groundwater monitoring. These results were consistent with the survival experiments, indicating that *E. coli* survived for much longer than Campylobacter in an unsaturated alluvial gravel media. 

Sinton et al. [52] found that the transport velocity of *E. coli* cells was greater than that of *B. subtilis* endospores, which in turn was greater than that of phages. They proposed that these results could be explained by the theory of pore size exclusion, according to which larger particles are preferentially transported through the larger interconnected pores, where water velocities are higher, and prevented from passing through smaller pores, which are available to smaller particles and dissolved chemicals.

Naclerio et al. [53] detected faecal enterococci (including *Enterococcus faecalis*) in springs fed from a fractured aquifer more frequently than in thermotolerant coliforms (mainly *E. coli*) because of the different decay rates of the two groups of microorganisms in groundwater. They compared the retention of thermotolerant coliforms and enterococci within the topsoil and the aquifer and concluded that the latter were a more reliable indicator of the microbial contamination of groundwater than thermotolerant coliforms.

## 4. Discussion and Conclusions

In this paper we reviewed a range of field-scale investigations of the factors that affect the microbial contamination of groundwater. We selected 24 studies published between 2003 and 2022 that considered various factors involved in the transport of microorganisms from the surface to the subsurface, which could promote or contrast the risks associated with waterborne diseases that result from using unhealthy groundwater for irrigation or drinking purposes. 

**Figure 3:**
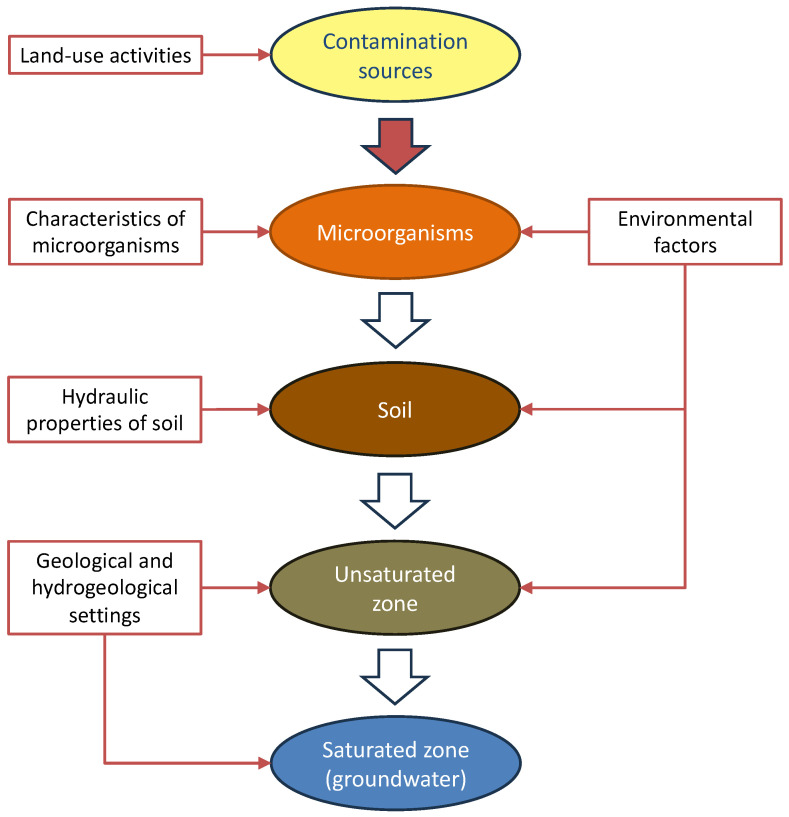
General scheme for microbial contamination of groundwater.

The selected literature indicates that the general scheme for microbial contamination of groundwater (Figure 3) is as follows: Microorganisms are released onto the soil by several anthropic activities. They first infiltrate the soil and then cross the unsaturated zone of the aquifer, before eventually reaching the saturated zone and contaminating the groundwater. During these phases, microorganisms are subject to the influence of various factors, including land-use activities, environmental conditions, the hydraulic properties of soil, and geological and hydrogeological settings. These factors influence the initial concentration, growth, removal, or hindrance of microorganisms in groundwater, with varying degrees of effectiveness. Additionally, the intrinsic characteristics of microorganisms can influence their ability to survive in the underground environment and interact with subsoil components.

The studies reported many land-use activities, such as septic tanks [58], raw or treated wastewater discharge [52,60,65,67,69,72], OSWTS [63], municipal solid waste landfills [68], manure applications [50,56,61,64], and grazing on pastures [51,53,54,57,59], as factors determining the microbial contamination for soil, subsoil, and groundwater.

Once released onto the surficial layer of soil, the microorganisms may decrease in number because of adverse environmental conditions and predation by other microbial species, which in turn depended on the environmental temperature [60]. Of the environmental factors studied, rainfall was associated with increases in the bacterial contamination of groundwater. Rainfall caused a decrease in salinity in coastal aquifers and a corresponding increase in bacterial survival [73], and promoted microbial transport towards groundwater in internal aquifers [51,54,55,57,66,70,71]. 

The soil moisture conditions were reported to have a strong effect on the rate of microorganism transport into the unsaturated portion of the subsoil. Specifically, the microorganism transport rate was higher in saturated conditions than unsaturated conditions, and the rate decreased as the moisture value decreased [50,56,61]. 

The unsaturated zone is a layer of the subsoil in which the main microbial removal processes take place, therefore its nature, structure, thickness, and geological and hydraulic characteristics could influence the transport of microorganisms [74]. Comparisons showed that the groundwater contamination was higher (1) when the unsaturated zone was thin rather than thick [58,65,67], (2) in permeable aquifers, such as fractured or karst aquifers, than in less permeable aquifers [64,72], and (3) in aquifers consisting mainly of macropores rather than micropores [50,52,64]. The results of numerous field observations regarding the effect of the unsaturated zone on the microbial contamination of groundwater were corroborated by laboratory-scale column studies. In particular, bacterial retention was found to be higher in unsaturated flow conditions than in saturated conditions [75], and to decrease in the medium that exhibited larger pores [76].

The size, interactions, and resistance of different microbial species determine their transport velocity and survival in the subsoil. In particular, larger microbial forms, such as *E. coli* cells, exhibited a greater transport rate than smaller ones, such as *B. subtilis* endospores and phages [52]. This phenomenon was previously reported in other studies [77,78], and can be explained by the theory of pore size exclusion, whereby larger particles exhibit higher transport velocities because their preferential pathway through larger porous media.

*E. coli* was the most frequently utilized microorganism among the analysed studies to describe the microbial transport from surface to groundwater. However, enterococci appeared to be more resistant to the different conditions of the underground environment than thermotolerant coliforms and so, of the two, were suggested as a better indicator for assessing pathogen survival in groundwater [53]. Several studies confirmed the greater environmental resistance of enterococci compared to coliforms. Byappanahalli et al. [79] observed that enterococci exhibited greater tolerance to salinity and disinfection than faecal coliforms and *Escherichia coli* in aquatic environments. Moriarty et al. [80] demonstrated that the concentration of enterococci in pastures contaminated by goose faeces decreased more slowly than coliforms in both summer and winter. Even in groundwater, enterococci demonstrated a slower inactivation rate than faecal coliforms, with a rate comparable to that of viruses [81].

The studies analysed in this review may have limitations because of the difficulties associated with field-scale studies that involve complex environmental systems. For example, the levels of microbial contamination detected in groundwater may result from the interactions of various factors some of which may be not included in the study design. As such, laboratory-scale experiments carried out under controlled conditions may provide more useful evidence about the role of a single factor or the interactions between two or multiple factors. The results of field and laboratory experiments were only integrated in five of the studies selected for this review [51,53,57,61,62]. 

In addition, studies that compare two or more different scenarios, with some conditions kept constant, could provide more useful results. Of the studies selected, most considered a single hydrogeological setting, and only one [72] compared the effects of two similar impacts in two different aquifers. Further, most studies focused on microorganisms and few studied viruses, despite the high prevalence of waterborne diseases of viral etiology. Finally, only a few studies were conducted in areas with high incidences of waterborne diseases.

In summary, groundwater represents a valuable source of water supply for human needs and for this reason, it should have more protection from chemical and microbiological contamination than surface water. However, its protection cannot be guaranteed, and, in certain conditions, groundwater could provide a pathway for pathogens that cause waterborne diseases. Studies of the conditions and factors that render groundwater unhealthy are important as they will support the development of guidance for appropriate management of anthropic pressures and the assessment of the health risks related to the use of the water resource. The in-field studies showed that microbial contamination of groundwater depended on complex interactions between human activities and the geological and hydraulic characteristics of the media that the water passed through, such as the moisture condition, and the characteristics and viability of the microorganisms, which in turn depended on the environmental conditions and presence of predatory species.

Future studies should integrate both field-scale and laboratory-scale experiments and should evaluate the behaviour of different microbial categories, particularly viruses, under different environmental and hydrogeological conditions. The overall aim should be to identify a set of indicators that represent the health risks from using the groundwater for drinking or irrigation purposes. Furthermore, geographical areas with a greater incidence of waterborne diseases should receive more attention than those with a low incidence, so that guidelines can be implemented to ensure the appropriate management of anthropic pressures and water resources intended for human consumption.

## Figures and Tables

**Table 1:** Field-scale studies published between 2003 and 2022 selected for the review and information regarding geographical area, contamination sources, environmental domains, target microorganism, and study design of each study.

Study	Location	Contamination Source	Environmental Domain	Target Microorganisms	Study Design
Cheng et al. [73]	China	Polluted seawater	Saturated porous aquifer	*E. coli*	Monitoring of groundwater
De Giglio et al. [72]	Italy	Effluent from WWTP	Saturated porous and karst aquifers	*E. coli*, enterococci, *Cl. perfringens*, enteric viruses (a)	Monitoring of effluent and groundwater
de Lambert et al. [71]	USA	Urban stormwater	Saturated and unsaturated porous aquifer	*E. coli*, total coliforms, Group A Rotavirus	Monitoring of cisterns, leachate and groundwater
Lorette et al. [70]	France	Punctual infiltration from surface runoff	Saturated karst aquifer	*E. coli*, total coliforms, Enterococcus	Monitoring of spring water
Marshall et al. [69]	Canada	Wastewater	Saturated fractured aquifer	*E. coli*, total coliforms	Monitoring of groundwater
Aromolaran et al. [68]	Nigeria	MSW dumpsite	Saturated fractured aquifer	Faecal coliforms, total coliforms	Monitoring of MSW leachate and groundwater
Weldeyohannes et al. [67]	Canada	Wastewater	Saturated porous aquifer	*E. coli*	Monitoring of wastewater and groundwater
Sreekala et al. [66]	India	Percolation following rainfall	Porous and fractured aquifers	*E. coli*, faecal coliforms, total coliforms	Monitoring of groundwater
Elkayam et al. [65]	Israel	Effluent from WWTP	Saturated and unsaturated porous aquifer	*E. coli*, faecal coliforms, faecal streptococci, Human viruses (b)	Monitoring of effluent, groundwater, and recovery water
Arnaud et al. [64]	Canada	Manure	Unsaturated porous and saturated fractured aquifer	*E. coli*, total coliforms	Sampling of soil, manure, and groundwater
Donohue et al. [63]	Ireland	Effluent from OSWTP	Saturated porous aquifer	*E. coli*, faecal coliforms	Monitoring of groundwater
Russel et al. [62]	USA	Contaminated beach sands	Saturated and unsaturated porous aquifer	Enterococci	Sampling of sand, leachate, and groundwater. Laboratory experiments
Unc et al. [61]	USA	Manure on irrigated fields	Saturated and unsaturated porous aquifer	*Enterococcus* spp.	Sampling of soil and groundwater. Laboratory experiments
Motz et al. [60]	Canada	Effluent from WWTP	Unsaturated porous aquifer	*E. coli*	Sampling of soil and pore water
Goeppert and Goldscheider [59]	Austria/Germany	Cattle pasturing	Saturated porous aquifer	*E. coli*, enterococci	Monitoring of spring water
Katz et al. [58]	USA	Septic tank	Saturated karst aquifer	Faecal coliforms, Enteroviruses	Sampling of septic tank effluent, soil, and groundwater
Close et al. [57]	New Zealand	Cattle pasturing	Unsaturated porous aquifer	*E. coli*, Faecal coliforms, *C. jejuni*, *B. subtilis*	Transport experiment (lysimeters) and monitoring of groundwater. Survival experiments
Mosaddeghi et al. [56]	Iran	Manure	Clay loam soil	*E. coli*	Sampling of soil and leachate (or soil solution)
Pronk et al. [55]	Switzerland	Percolation following rainfall	Unsaturated Karst aquifer	*E. coli*, enterococci	Monitoring of water dripped from a fracture
Levison and Novakowski [54]	Canada	Cattle pasturing	Saturated fractured aquifer	*E. coli*	Monitoring of groundwater
Naclerio et al. [53]	Italy	Cattle pasturing	Saturated fractured aquifer	Thermotolerant coliforms, faecal streptococci	Monitoring of spring water. Laboratory experiments
Sinton et al. [52]	New Zealand	Effluent from WWTP	Saturated porous aquifer	Faecal coliforms, *B. subtilis*, F-RNA phages, *E. coli* J6-2, Somatic coliphage ØESR1	Sampling of effluent and groundwater
Celico et al. [51]	Italy	Cattle pasturing	Saturated fractured aquifer	Faecal coliforms, faecal enterococci	Monitoring of spring water. Laboratory experiments
Unc and Goss [50]	Canada	Manure	silty loam and sandy loam soil	*E. coli*, total coliforms	Sampling of manure and soil

(a): adenovirus, norovirus genogroup I and II, enterovirus, hepatitis A virus, hepatitis E virus, and rotavirus; (b): adenovirus, enterovirus, norovirus, parechovirus.

**Table 2:** Comparative table on the microbiological parameters of contaminated waters.

Study	Source	Target Microorganisms	Concentration in Source	Concentration in Groundwater
Cheng et al. [73]	Polluted seawater	*E. coli*	n.r.	39.7 cfu 100 mL^−1^ (a)
De Giglio et al. [72]	Effluent from WWTP	*E. coli*	6–893 cfu/100 mL (a)	<1 cfu/100 mL (a)
		Enterococci	5–16.1 cfu/100 mL (a)	<1 cfu/100 mL (a)
		*C. perfringens*	3–5 cfu/100 mL (a)	<1–2 cfu/100 mL (a)
		Enteric viruses (b)	26.7% (c)	6.7% (c)
de Lambert et al. [71]	Urban stormwater (cistern)	*E. coli*	2–190 MPN/100 mL	0
		Total coliforms	610–24,000 MPN/100 mL	<1–2400 MPN/100 mL
		Group A rotavirus	2/8 (c)	0/8 (c)
Lorette et al. [70]	Punctual infiltration from surface runoff	*E. coli*	n.r.	10–300 cfu/100 mL (d)
		Total coliforms	n.r.	20–600 cfu/100 mL (d)
		Enterococcus	n.r.	2–54 cfu/100 mL (d)
Marshall et al. [69]	Wastewater	*E. coli*	n.d.	10^1^–10^4^ cfu/100 mL
		Total coliforms	n.d.	10^1^–10^4^ cfu/100 mL
Aromolaran et al. [68]	MSW leachate	Faecal coliforms	40 × 10^4^ cfu/mL	n.r.
		Total coliforms	87 × 10^4^ cfu/mL	n.r.
Weldeyohannes et al. [67]	Wastewater	*E. coli*	10^4^–10^5^ MPN 100 mL^−1^	<3 × 10^2^ 100 mL^−1^
Sreekala et al. [66]	Percolation following rainfall	*E. coli*	n.r.	n.r.
		Faecal coliforms	n.r.	n.r.
		Total coliforms	n.r.	n.r.
Elkayam et al. [65]	Effluent from WWTP	*E. coli*	240 × 10^3^ cfu/mL	<1–1 cfu/mL
		Faecal coliforms	160 × 10^3^ cfu/mL	<1 cfu/mL
		Faecal streptococci	9.4 × 10^3^ cfu/mL	<1 cfu/mL
		Human viruses (e)	13,000–115,000 copies/1000 L	<1–1 copies/1000 L
Arnaud et al. [64]	Liquid manure	*E. coli*	4.6 × 10^4^ cfu/100 mL (a)	60–>300 cfu/100 mL (a)
		Total coliforms	6.6 × 10^4^ cfu/100 mL (a)	< 5 cfu/100 mL
Donohue et al. [63]	Effluent from OSWTP	*E. coli*	n.r.	<1–11,190 MPN/100 mL
		Total coliforms	n.r.	<1–241,960 MPN/mL
Russel et al. [62]	Seawater	Enterococci	146 MPN/100 mL	n.r.
Unc et al. [61]	Manure slurry	*Enterococcus* spp.	3 × 10^6^ cfu/100 mL	0.04 cfu/100 mL
Motz et al. [60]	Effluent from WWTP	*E. coli*	2.5 × 10^4^ MPN/100 (a)	0 MPN/100 mL (f)
Goeppert and Goldscheider [59]	Cattle pasturing	*E. coli*	n.r.	0–65 cfu/100 mL (d)
		Enterococci	n.r.	0–24 cfu/100 mL (d)
Katz et al. [58]	Septic tank effluent	Faecal coliforms	1.2 × 10^5^–7.8 × 10^6^ cfu/100 mL	0–6.0 cfu/100 mL
		Enteroviruses	6/8 (c)	0/0 (c)
Close et al. [57]	Cattle pasturing (transport)	Faecal coliforms	n.r.	2–1600 cfu 100 mL^−1^ (f)
		*Campylobacter jejuni*	n.r.	<0.5–4 MPN 100 L^−1^ (f)
		*B. subtilis*	n.r.	<1–1180 cfu 100 mL^−1^ (f)
	Cattle pasturing (monitoring)	*E. coli*	n.r.	1−6 cfu 100 mL^−1^
		*Campylobacter jejuni*	n.r.	3/419 (0.7%) (c)
Mosaddeghi et al. [56]	Manure—soil	*E. coli*	n.r.	n.r.
Pronk et al. [55]	Percolation following rainfall	*E. coli*	n.r.	0–17,000 cfu/100 mL (g)
		Enterococci	n.r.	0–300 cfu/100 mL (g)
Levison and Novakowski [54]	Cattle pasturing	*E. coli*	n.r.	0–900 counts/100 mL
Naclerio et al. [53]	Cattle pasturing	Thermotolerant coliforms	n.r.	0–898 cfu 1000 mL^–1^ (d)
		Faecal enterococci	n.r.	0–2216 cfu 1000 mL^–1^ (d)
Sinton et al. [52]	Effluent from WWTP	Faecal coliforms	n.r.	2.00 × 10^3^
		*Bacillus subtilis*	n.r.	n.r.
		F-RNA phages	n.r.	5.0
		*E. coli J6-2*	n.r.	4.40 × 10^4^
		Somatic coliphage ØESR1	n.r.	1.09 × 10^5^
Celico et al. [51]	Cattle pasturing	Faecal coliforms	n.r.	0–100 cfu/100 mL (d)
		Faecal enterococci	n.r.	0–94 cfu/100 mL (d)
Unc and Goss [50]	Manure (liquid swine)	Total coliforms	7.14 log_10_ cfu 100 mL^−1^ (a)	0-16% (c) (h)
		*E. coli*	6.38 log_10_ cfu 100 mL^−1^ (a)	
	Manure (solid cow)	Total coliforms	8.73 log_10_ cfu 100 g^−1^ (a)	0-12% (c) (h)
		*E. coli*	8.55 log_10_ cfu 100 g^−1^ (a)	

n.r.: not reported; cfu: colony-forming unit; MPN: most probable number; (a): mean concentration; (b): adenovirus, norovirus genogroup I and II, enterovirus, hepatitis A virus, hepatitis E virus, and rotavirus; (c): positive samples; (d): spring water; (e): adenovirus, enterovirus, norovirus, parechovirus; (f): lecheate from lysimeter; (g): water dripped form a fracture; (h): soil at 1.00 m depth.

**Table 3:** Main findings of the studies selected for the review.

**Study**	**Main Findings**
Cheng et al. [73]	Rainfall was responsible for a decrease in salinity and consequently for an increase in *E. coli* survival in a beach aquifer.
De Giglio et al. [72]	A porous vadose zone was better at retaining the microbial contaminants contained in the effluent from a WWTP than a karst vadose zone.
de Lambert et al. [71]	Microbial concentrations varied across sampling events and rainfall events were one driver of this variability.
Lorette et al. [70]	Point source infiltration from allochthonous turbidity with associated organic particles and bacteria, generated by surface runoff.
Marshall et al. [69]	The impact of wastewater contamination derived from a sparse distribution of small, aging septic systems is a serious concern in fractured sedimentary bedrock settings with thin overburden.
Aromolaran et al. [68]	The leachate from an MSW dumpsite contained a variety of microorganisms, including enteric bacteria, that were responsible for the microbial contamination of a fractured aquifer.
Weldeyohannes et al. [67]	The detection of *E. coli* in groundwater significantly declined when the unsaturated zone thickness was equal or greater than 0.9 m.
Sreekala et al. [66]	There were significantly fewer total coliforms in the pre-monsoon season than during the monsoon and post monsoon seasons in sedimentary and hard crystalline aquifers.
Elkayam et al. [65]	The microbial contamination decreased by more than four orders of magnitude in the unsaturated zone.
Arnaud et al. [64]	*E. coli* levels decreased significantly when the thickness of the unsaturated zone exceeded 0.9 m.
Donohue et al. [63]	Microbiologically contaminated effluent from an OSWTS that was discharged to a thin layer of glacial till subsoil that rested on a poorly productive greywacke/shale bedrock aquifer had the capacity to cause gross contamination of groundwater in the immediate vicinity of the percolation area.
Russel et al. [62]	The infiltration of seawater into the unsaturated zone promoted the transport of enterococci from polluted sands and the contamination of groundwater.
Unc et al. [61]	In soils where manure had been applied, transport in the unsaturated zone was more likely to involve rapid transport of enterococcus spp. from the surface in irrigation water than the mobilization of bacteria in the unsaturated zone.
Motz et al. [60]	Seasonal variations in temperature could influence the metabolic rate of both *E. coli* and their predators and the *E. coli’s* survival.
Goeppert and Goldscheider [59]	The temporal variability in the faecal indicator bacteria in the springs reflected the different land-use activities in the recharge areas.
Katz et al. [58]	Faecal indicators and enteric viruses were detected at higher levels in the shallowest portion of a limestone karst aquifer, overlayed by a thin layer of sands and clays.
Close et al. [57]	Spray irrigators resulted in negligible transport of microbes through the soil and unsaturated zone. *E. coli* and Campylobacter in shallow groundwater were mainly detected during periods of heavy rainfall. Faecal coliform bacteria survived much longer in the unsaturated alluvial gravel media than Campylobacter.
Mosaddeghi et al. [56]	The degree of water saturation and sampling depth had significant effects on the bacterial transport: the rates of bacterial transport were greater in the saturated flow than in the unsaturated flow.
Pronk et al. [55]	The exclusion processes of faecal bacteria, which are known from saturated porous media, also occur in the soil and unsaturated zone of a karst aquifer.
Levison and Novakowski [54]	The contaminant concentrations in bedrock aquifers with minimal overburden were variable and unpredictable because of periodic upgradient sources, dilution from recharge, and heterogeneous flow systems
Naclerio et al. [53]	Enterococci were a better indicator of faecal contamination than thermotolerant coliforms because of the different decay and retention characteristics of the two microorganisms within the topsoil and the aquifer.
Sinton et al. [52]	Most of the enteric microorganisms percolating from the soil surface were removed in the micropores, and between 1% and 5% reached the groundwater via macropore flow.
Celico et al. [51]	The transport of faecal bacteria in the subsurface was strongly influenced by precipitation and soil in small, extensively fractured limestone aquifers.
Unc and Goss [50]	Shallow water tables underlying soils in manured fields containing a significant proportion of macropores were particularly vulnerable to contamination by faecal bacteria. Moist soil conditions facilitated the microbial contamination of groundwater.

## Data Availability

No new data were created or analysed in this study. Data sharing is not applicable to this article.
